# Alterations in regional homogeneity of resting-state brain activity in internet gaming addicts

**DOI:** 10.1186/1744-9081-8-41

**Published:** 2012-08-18

**Authors:** Guangheng Dong, Jie Huang, Xiaoxia Du

**Affiliations:** 1 Department of Psychology, Zhejiang Normal University, 688 of Yingbin Road, Jinhua city, Zhejiang Province, P.R.China; 2 Department of Physics, Shanghai Key Laboratory of Magnetic Resonance, East China Normal University, Shanghai, P.R.China

**Keywords:** Internet addiction disorder, Internet gaming addiction, Resting-state fMRI

## Abstract

**Backgrounds:**

Internet gaming addiction (IGA), as a subtype of internet addiction disorder, is rapidly becoming a prevalent mental health concern around the world. The neurobiological underpinnings of IGA should be studied to unravel the potential heterogeneity of IGA. This study investigated the brain functions in IGA patients with resting-state fMRI.

**Methods:**

Fifteen IGA subjects and fourteen healthy controls participated in this study. Regional homogeneity (ReHo) measures were used to detect the abnormal functional integrations.

**Results:**

Comparing to the healthy controls, IGA subjects show enhanced ReHo in brainstem, inferior parietal lobule, left posterior cerebellum, and left middle frontal gyrus. All of these regions are thought related with sensory-motor coordination. In addition, IGA subjects show decreased ReHo in temporal, occipital and parietal brain regions. These regions are thought responsible for visual and auditory functions.

**Conclusions:**

Our results suggest that long-time online game playing enhanced the brain synchronization in sensory-motor coordination related brain regions and decreased the excitability in visual and auditory related brain regions.

## Background

Internet addiction disorder (IAD) is usually defined as the inability of an individual to control his or her use of the internet. This inability will eventually cause psychological, social, and/or work difficulties [1-4]. As the world’s fastest growing ‘addiction’, IAD has been considered as a serious public health issue [1,5,6]. Although significant prevalence estimations and associations with adverse consequences have been addressed [1,7-11], few studies focused on the neurobiological underpinnings of IAD [12-14]. Thus, investigations on the neurobiological basis of IAD are necessary and important. Discovery of the biomarkers of IAD will provide valuable information on unraveling the underpinnings of IAD and on assessing proper treatment strategies.

IAD is also considered as a behavioral addiction and may share similar neuropsychological (i.e., development of euphoria, craving, and tolerance) and personality characteristics with other addictions [15], especially behavioral addiction, for example, gambling addiction [16]. Internet addiction consists at least three subtypes: Internet gaming addiction (IGA), sexual preoccupations, and email/text messaging [12,14]. Comparing to other subtypes of IAD, internet gaming addiction, the most important subtype of IAD, exhibits some specific features such as role-playing in the virtual world. Few studies have addressed the brain functional changes in people who are addicted to the internet games. Previous studies showed that mental disorders could change the spontaneous activity in the brain [17-19]. Researchers believed that internet addiction is an impulse disorder or is at least related to it [20]. Thus, IGA may share similar neuropsychological and personality characteristics with other mental disorders or behavioral addictions [15,16]. Potential changes in brain are expected in IGA patients by analogy with other mental disorders.

Resting-state functional MRI has been increasingly used to investigate the integration of neural activities at a resting state when no task is performed [21]. Although resting-state fMRI is a relatively new technique, many exciting findings have been reported in the past several years (for a review see [22]). Nowadays, this technique has been successfully used to detect abnormal functional integration in some mental disorders [23,24]. Regional homogeneity (ReHo) is a widely used method in resting-state fMRI studies [21,25,26]. ReHo measures the functional coherence of a given voxel with its nearest neighbors, which can be used to evaluate resting-state brain activities based on the hypothesis that significant brain activities would more likely occur in clusters than in a single voxel [27]. ReHo index could be regarded as a measurement for investigating human brain activities in the resting state and may be useful for revealing the complexity of human brain function [28,29].

Online game playing requires players staring at the computer screen and enduring the sound of the game for a long time. Previous studies have shown that noise could cause hearing problem [30-33], and long-time hypertension visual attention could cause serious visual acuity decrease or vision loss [34]. Thus, the long-time game playing might impair the visual or hearing functions and these changes could be detected in relevant brain regions. In this study, we hypothesized that long-time online game playing changed the brain functions in the regions that are related with vision or hearing. To address this issue, we employed resting-state fMRI to examine the spontaneous fluctuations of brain activities in IGA subjects in this study.

## Methods and procedure

### Subject

We examined a group of 15 (16–1) male IAD participants (i.e., male, right handed, non-smokers) aged 24.2 ± 3.5 years (mean ± SD) and a group of 14 (15–1) age matched, healthy males aged 24.6 ± 3.8 years (mean ± SD) (i.e., right handed, non-smokers). Our study focused on male subjects because the prevalence of IAD in men is much higher than that in women [35]. IAD subjects were recruited through advertisements. All participants underwent structured psychiatric interviews (M.I.N.I.) performed by an experienced psychiatrist with an administration time of approximately 15 minutes. The MINI was designed to meet the need for a short but accurate structured psychiatric interview for multicenter clinical trials and epidemiology studies [36]. All participants were free of Axis I psychiatric disorders listed in M.I.N.I. Depression was further assessed using the Beck Depression Inventory [37]. Anyone who scored more than 5 would be excluded from our study.

All IAD subjects had a diagnosis of Young’s online internet addiction test [38], which consists of 20 items associated with online internet use including psychological dependence, compulsive use, and withdrawal, as well as related problems in school or work, sleep, family, and time management. For each item, a graded response is selected from 1 = “Rarely” to 5 = “Always”, or “Does not Apply”. The IAT is proved to be a valid and reliable instrument that can be used in classifying IAD [39,40]. People who scored more than 50 were considered to experience occasional or frequent problems because of the internet. Those who scored more than 80 were considered to have significant problems in their lives [38]. In the present study, the threshold cut-off we used is 80 in the internet addiction test. Estimates of the size of the group of ‘addicted gamers’ are defined by applying various cut-off points to scales measuring symptoms of internet addiction [41,42]. In present study, we added some specific limitations on the established measures of internet addiction, such as ‘you spend most of your time playing online games (>80%)’.

The controls were also measured with the same process. Controls scored lower than 20 in Young’s test (16.3 ± 4.3 mean ± SD). All participants were medication free and were instructed not to use any substance of abuse, including coffee, on the day of the scan. No participant had previous experience with cocaine or marijuana. The human investigation committee of Zhejiang normal university approved this research. All subjects signed a written informed consent.

### Scanning process

The ‘resting state’ was defined as no specific cognitive task during the fMRI scan. Participants were required simply to keep still, close their eyes and not to think of anything systematically [27,28]. Magnetic resonance imaging scanning MRI data were acquired using a Siemens Trio 3 T scanner (Siemens, Erlangen, Germany) in East-China Normal University. Participants lay supine with head snugly fixed by belt and foam pads to minimize head movement. The resting-state functional images were acquired by using an echo-planar imaging sequence with the following parameters: interleaved, 33 axial slices, thickness = 3.0 mm, in-plane resolution = 64* 64, repetition time = 2000 ms, echo time = 30 ms, flip angle = 90, field of view = 220* 220 mm, 240 volumes (8 min). Structural images were collected using a T1-weighted three-dimensional spoiled gradient-recalled sequence was acquired covering the whole brain (176 slices, repetition time = 1700 ms, echo time TE = 3.93 ms, slice thickness = 1.0 mm, skip = 0 mm, flip angle = 15, inversion time 1100 ms, field of view = 240*240 mm, in-plane resolution = 256* 256).

### Preprocessing of image data

Preprocessing and ReHo process were using the software DPARSF (Data Processing Assistant for Resting-State fMRI. http://www.restfmri.net), a MATLAB toolbox for ‘pipeline’ data analysis of resting-state fMRI [43,44]. DPARSF is based on some functions in SPM and REST. The main pre-processing steps and parameters are: The first 10 volumes of each functional time series were discarded for participant adaptation to the scanning. Then, the image preprocessing, including slice timing, head motion correction and spatial normalization were conducted. After the procedure of head motion correction, the values for translation (mm) and rotation (degrees) were obtained at each time point in six parameters (three for translation and three for rotation) for each participant. Participants (1 from IAD group and 2 from control group) with head motion more than 2.0 mm maximum displacement in any direction of x, y, and z or 1° of any angular motion throughout the course of scan were excluded from further analysis. The fMRI data were temporally band-pass filtered (0.01-0.08 Hz) to reduce the low-frequency drift and physiological high-frequency respiratory and cardiac noise. Then the time-series of images of each subject were motion-corrected using a least squares approach and a six-parameter (rigid body) linear transformation. Individual ReHo map was generated by calculating Kendall’s coefficient concordance (with value from 0 to 1) of time series of a given voxel with those of its nearest neighbors (26 voxels) in a voxel-wise way. Spatial smoothing was conducted on the ReHo maps with a Gaussian kernel of 4 * 4 * 4 mm^3^ full-width at half-maximum. The intracranial voxels were extracted to make a mask [43]. For standardization purposes, each individual ReHo map was divided by its own mean ReHo within the mask.

## Statistical analyses

### ReHo analysis

Two-sample t-test was performed on the normalized individual ReHo maps. The resultant statistical map was set at a combined threshold of *p*_*FDR*_ < 0.05 and a minimum cluster size is 10 voxels (270 mm^3^).

### Correlation analysis

We calculated the correlation between IAT scores and the averaged peak values in all these survived clusters after correction in brainstem, inferior parietal lobule, left posterior cerebellum, and left middle frontal gyrus in IAD subjects. In addition, we also calculated the correlation between IAT score and the averaged peak values in all these survived clusters after correction in temporal, occipital and parietal brain regions in IAD subject.

### Functional connectivity analysis

The inferior parietal lobule of the left hemisphere lies at the junction of the auditory, visual, and somatosensory cortexes. Thus, we selected this region as the seed region. We calculated the functional connectivity between the seed and other brain regions in the ANOVA analysis showing significant ReHo difference between the two groups (IAD-Controls).

## Results

### ReHo results

**Figure 1:**
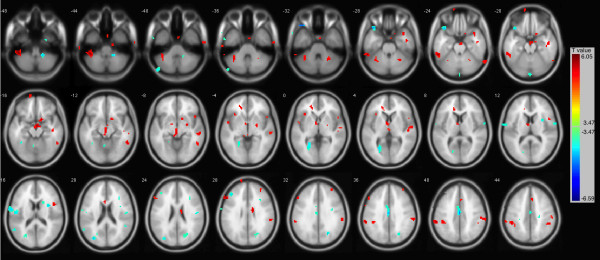
**Increased and decreased ReHo in IAD when comparing to healthy controls.** Voxel size = 3*3*3. *p* < 0.05 FDR corrected.

As shows in Table 1 and Figure 1, the IAD subjects displayed significantly increased ReHo in brainstem (left and right), inferior parietal lobule (left and right), left posterior cerebellum, and left middle frontal gyrus when comparing with healthy controls. In addition, significantly decreased ReHo was discovered in left superior and inferior temporal gyrus, left occipital lobe and parietal lobe.

**Table 1:** Brain areas of ReHo difference between two groups (IAD - Controls)

**Main areas**	**Hemisphere**	**Peak MNI coordinates (x, y , z)**	**Voxels**	**Peak *t*value**
*Increased ReHo in IAD*				
Right Brainstem	R	3, -6, -15	23	5.0501
Left Brainstem	L	−3, -21, -12	16	3.6839
Inferior Parietal Lobule	R	63, -30, 33	19	3.5916
Inferior Parietal Lobule	L	−60, -36, 39	18	4.3319
Cerebellum Posterior Lobe	L	−48, -33, -51	47	4.7067
Middle Frontal Gyrus	L	−60, 27, 30	63	4.3593
*Decreased ReHo in IAD*				
Inferior Temporal Gyrus	L	−54, -42, -24	19	−4.3287
Superior Temporal Gyrus	L	−39, 33 -33	25	−5.2271
Occipital Lobe, limbic lobe	L	−24, -63, 3	20	−3.9607
Parietal Lobe (Postcentral)	L	−63 ,-6, 15	19	−3.8942
Middle Cingulate Gyrus	L	0, -15, 39	23	−4.9526

Voxel size = 3*3*3 mm. *p* < 0.05 FDR corrected and at least 10 voxels.

### Correlation results

**Figure 2:**
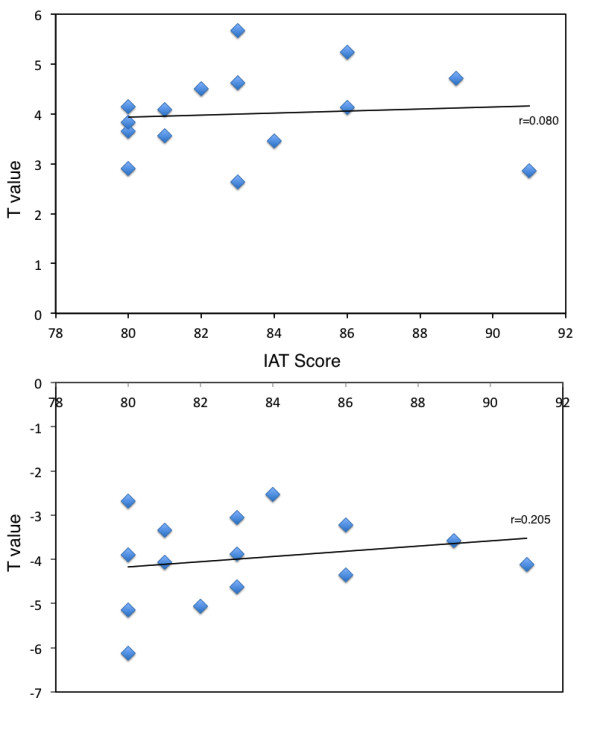
Correlation between the mean peak values survived after correction (hyper-activation) and IAT scores (upper); Correlation between the mean peak values survived after correction (hypo-activation) and IAT scores (upper) (bottom).

The correlation between IAT scores and the averaged peak values in all these survived clusters after correction in brainstem, inferior parietal lobule, left posterior cerebellum, and left middle frontal gyrus in IAD subjects is 0.080. The correlation between IAT score and the averaged peak values in all these survived clusters after correction in temporal, occipital and parietal brain regions in IAD subject is 0.205. Maybe because there are only 15 IAD subjects in present study, no significant correlation was found among these factors (Figure 2).

### Functional connectivity results

Table 2 shows the functional connectivity results. Although no significant difference was found between the IAD group and healthy controls, but the results still can provide some support to our speculations in this study (Table 2).

**Table 2:** Differences of functional connectivity in the brain regions survived in ReHo analysis between IAD and HC (Seed: inferior parietal lobule in the left hemisphere)

**Location**	**Hemisphere**	**Peak MNI coordinates (x, y , z)**	**T value (IAD-Control)**	**P value**
Right Brainstem	R	3, -6, -15	3.13	*p* > 0.05
Left Brainstem	L	−3, -21, -12	2.37	*p* > 0.05
Inferior Parietal Lobule	R	63, -30, 33	3.96	*p* > 0.05
Cerebellum Posterior Lobe	L	−48, -33, -51	1.71	*p* > 0.05
Middle Frontal Gyrus	L	−60, 27, 30	0.89	*p* > 0.05
Inferior Temporal Gyrus	L	−54, -42, -24	1.38	*p* > 0.05
Superior Temporal Gyrus	L	−39, 33 -33	1.84	*p* > 0.05
Occipital Lobe, limbic lobe	L	−24, -63, 3	0.69	*p* > 0.05
Parietal Lobe (Postcentral)	L	−63 ,-6, 15	2.94	*p* > 0.05
Middle Cingulate Gyrus	L	0, -15, 39	1.52	*p* > 0.05

## Discussion

Decreased or increased ReHo in brain suggest neural functions in certain regions are less or more synchronized [27]. In this study, ReHo analysis depicted several brain areas, where local BOLD signal coherence are different in IGA subjects when comparing with that of healthy controls.

### Enhanced sensory-motor coordinate ability

Enhanced ReHo was found in brainstem, inferior parietal lobule, left posterior cerebellum, and left middle frontal gyrus. All these areas are responsible for coordinating motor-sensory ability to some degrees. The brainstem serves as a conduit for many ascending and descending pathways. It is an extremely important part of the brain as the nerve connections of the motor and sensory systems from brain to the rest of the body [45,46]. The cerebellum is a region of the brain that plays an important role in motor control [47]. The cerebellum does not initiate movement, it receives input from sensory systems and from other parts of the brain, and integrates these inputs to fine tune motor activity [48]. The inferior parietal lobule of the left hemisphere lies at the junction of the auditory, visual, and somatosensory cortexes, with which it is massively connected. In addition, the neurons in this lobule can process different kinds of stimuli (auditory, visual, sensorimotor, etc.) simultaneously [49,50]. Functional dissociation between the left and right middle frontal gyrus has been suggested in working memory, which is strongly related to the involved mental operations [51].

Considering the characteristics of IAD and the functions of the brain regions we discussed above, we may infer that IAD subjects have enhanced coordinate ability in motor and sensory systems. When playing online game, players need to build avatars in their virtual world [52]. They also need to compete or cooperate with other players to fight against some enemies. Their actions, such as fighting, killing, shooting, using all kinds weapons and so on, are complicated and challenging. Players need to control their avatars skillfully to accomplish their tasks. Thus, the online game playing requires players to coordinate several systems, including the sensory system, motor control, motor coordinate and information processing system. In addition, online internet games can not be completed in a set time because of the regular introduction of new contents, which require players to continue playing to ‘keep up’ with the game [53]. These games may be addictive because they are particularly good at inducing operant conditioning via variable-ratio reinforcement schedules (a highly effective conditioning paradigm [54]). From what we discussed above, we may infer that the long-term game playing enhanced coordinate ability among motor, sensory and information processing systems in brain.

### Impaired visual and auditory function

Consistent with our hypothesis, the IAD subjects show decreased ReHo in temporal, occipital and parietal brain regions when comparing with the healthy controls. These regions are thought to be responsible for visual and auditory functions. The superior temporal gyrus contains several important structures of the brain, including the location of the primary auditory cortex, which is responsible for the sensation of sound [55]. Inferior temporal gyrus is one of the higher levels of the ventral stream of audio and visual processing, associated with the representation of complex object features [56,57]. The occipital lobe is the visual processing center of the mammalian brain containing most of the anatomical region of the visual cortex [58,59]. The parietal lobe integrates sensory information from different modalities, particularly determining spatial sense and navigation [60-62].

The decreased ReHo in visual and auditory related brain regions might suggest the decreased synchronization in IGA subjects. Considering the features of IGA, we can infer that this may be the result of long-time game playing. The gaming process requires players to pay full attention to each subtle change in the screen, to endure the noisy sound of the game for a long time (usually more than 10 hours a day). The visual and auditory related brain regions have been stimulated for a long time, which made them hardly to be excited or have a decreased excitability. As we mentioned in the introduction section, long time hypertension of visual attention can impair subjects’ visual functions and noise can impair their hearing abilities [17-19]. Thus, we may infer that the long-time game playing (expose to visual and auditory stimuli) may impair player’s visual and auditory ability, which could be indexed by the decreased ReHo in this study.

## Limitation

Our results may sound more persuasive if we include a group of video game addicts as another control group. From comparisons between IGA subjects and video game addicts, we may find some specific features of IGA. In addition, if we had vision and hearing tests on the subjects before scan and take these variables as regressors in data analysis, we could get better scientific results. These issues will be emphasized in our future studies. Third, correlation and functional connectivity results cannot provide strong support to the conclusions about the impairment in visual auditory ability. Future studies should involve more participants to investigate this issue.

## Conclusions

From what we discussed above, we can conclude that long-time online game playing enhanced the sensory-motor coordinate ability and impaired participants’ visual and auditory functions. All these results can help policy-makers or individuals understand the influence of internet gaming addiction. First, this research can help us understand the mechanism of addiction, find if it shares characteristics with other types of addictions; Second, this research revealed the influence of internet games, especially its negative aspects to our brain mechanism.

## Competing interests

The authors do not have an affiliation with or financial interest in any organization that might pose a competing interest.

## Authors’ contributions

GD took part in designing the research and the manuscript preparation. JH analyzed the data. XD collected the data and took part in manuscript preparation. All authors read and approved the final manuscript.
